# Immunomodulatory effects of recombinant BCG expressing pertussis toxin on TNF-alpha and IL-10 in a bladder cancer model

**DOI:** 10.1186/1756-9966-27-78

**Published:** 2008-11-28

**Authors:** Daher C Chade, Ricardo C Borra, Ivan P Nascimento, Fabiola E Villanova, Luciana CC Leite, Enrico Andrade, Miguel Srougi, Kátia L Ramos, Priscila M Andrade

**Affiliations:** 1 Division of Urology, University of São Paulo, São Paulo, Brazil; 2 Ibirapuera University, São Paulo, Brazil; 3 Laboratory of Molecular Biotechnology IV – Butantan Institute, São Paulo, Brazil; 4 Laboratory of Medical Investigation (LIM55) of the Division of Urology, University of São Paulo, São Paulo, Brazil

## Abstract

**Background:**

Since successful treatment of superficial bladder cancer with BCG requires proper induction of Th1 immunity, we have developed a rBCG-S1PT strain that induced a stronger cellular immune response than BCG. This preclinical study was designed to compare the modulatory effects of BCG and rBCG-S1PT on bladder TNF-α and IL-10 expression and to evaluate antitumour activity.

**Methods:**

For Experiment I, the MB49 bladder cancer cell line was used in C57BL/6 mice. Chemical cauterization of the bladder was performed to promote intravesical tumor implantation. Mice were treated by intravesical instillation with BCG, rBCG-S1PT or PBS once a week for four weeks. After 35 days the bladders were removed and weighed. TNF-〈 and IL-10 cytokine responses were measured by qPCR. Experiment II was performed in the same manner as Experiment I, except the animals were not challenged with MB49 tumor cells. Results: rBCG-S1PT immunotherapy resulted in bladder weight reduction, compared to the BCG and control group. There were increases in TNF-α in the BCG-treated group, as well as increases in TNF-α and IL-10 mRNA in the rBCG-S1PT group.

**Conclusion:**

These data indicate a significant reduction of bladder tumor volume for the rBCG group, compared to the BCG and PBS groups. This suggests that rBCG could be a useful substitute for wild-type BCG and that the potential modulation between TNF-α and IL-10 cytokine productions may have therapeutic value.

## Background

Although the antitumor effects of the Bacillus Calmette Guerin vaccine (BCG) have long been demonstrated, its mechanism of action still raises many questions. In 1959, initial findings were reported of increased resistance to cancer induced by BCG [1]. Many studies have shown the relevance of this translational finding for bladder cancer. In 1976, a report by Morales indicated promising results in the treatment of superficial bladder cancer (SBC) with BCG [2]. More recently, Herr described BCG treatment as the most successful immunotherapy used for human tumors [3].

Following transurethral resection (TUR), BCG is considered an important coadjuvant in the treatment of superficial bladder cancer. Immunotherapy has also been superior to intravesical chemotherapy in patients with carcinoma *in situ *(CIS) [4]. BCG instillation has the main goal of decreasing bladder cancer recurrences and preventing tumor progression. Consequently, intravesical BCG is well established in the management of high grade Ta/T1 urothelial carcinoma, as well as CIS [5].

However, numerous obstacles are encountered when patients undergo intravesical BCG therapy. Treatment failure, including recurrence and progression, ranges from 40 to 70%, depending on factors such as tumor stage and histology grade [6]. In addition, a significant reason for concern is the high incidence of adverse events related to BCG immunotherapy. Complications include local or systemic side effects. The local adverse reactions occur in 27 to 95% of patients receiving immunotherapy, mostly causing irritative lower urinary tract symptoms. These expected mild reactions are rarely significant enough to interrupt the treatment. However, systemic side effects may occur, with fever (2–17%) being the most common severe adverse event. Considering both BCG treatment failure and the potential side effects, researchers have pursued other agents with similar antitumor activity, which are hoped to be more efficient and have lower morbidity [7].

Recombinant BCG technology has allowed the construction of immunotherapeutic agents aimed at specific targets. The objective is to stimulate the BCG-induced immune response that is directly related to the antitumor effect, inhibiting side reactions that do not participate in this process. This may be achieved by further elucidating the main steps responsible for the immunotherapeutic action in bladder cancer. Studies have demonstrated the importance of the local inflammatory response, which is characterized by an influx of leukocyte subpopulations, such as granulocytes, CD4 and CD8 T cells and NK cells, and granuloma formation. Following this cellular infiltration, cytokines characterized as part of the T helper types 1 and 2 (Th1 and Th2) immune response can be measured in the urine and blood of patients. Cytokines released by BCG stimulation and considered significant for the antineoplasic response are essentially those related to Th1 (IL-12, IL-2, TNF-α and IFN-γ), while a BCG inhibitory effect is related to the Th2 (IL-10) response [8]. In experimental studies the effectiveness of BCG against bladder cancer has been related to the presence of delayed-type hypersensitivity (DTH). Nadler *et al*. observed enhanced DTH when IL-10 was absent, either by antibody inhibition or through the use of IL-10-deficient (IL-10-/-) mice [9].

Nascimento *et al*. constructed a recombinant BCG (rBCG) expressing the genetically detoxified S1PT (rBCG-S1PT) fused with the signal sequence and under the control of the up-regulated *Mycobacterium fortuitum *^®^-lactamase promoter. It has been demonstrated that when compared to control BCG, rBCG-S1PT is able to elicit a Th1-driving effect on the immune response induced against mycobacterial proteins [10].

Therefore, we propose evaluation of the effectiveness on tumor reduction of rBCG-S1PT, compared to BCG and PBS; and evaluation of the immune response profile focusing on TNF-α (Th1) and IL-10 (Th2) in an orthotopic bladder cancer animal model [11,12].

## Methods

### Animals

Ninety female C57BL/6 mice aged six to eight weeks were provided by the Bioterism Center of the Medical Faculty of University of São Paulo, Brazil, and maintained at our animal care facility for one week prior to use. The mice were kept in a limited access area at a controlled room temperature, with food and water *ad libitum*. The mice were divided into groups for two different experiments (with or without bladder tumor implantation). All experiments were approved by the institution's ethical board.

### BCG

In all experiments, BCG (Moreau) and rBCG-S1PT, kindly provided by Dr. Luciana C.C. Leite (Butantan Institute), were prepared according to previously described procedures [10]. Briefly, BCG and rBCG-S1PT were cultured under uniform conditions in Middlebrook 7H9 supplemented with albumin dextrose-catalase broth and incubated at 37°C with 5% CO2 and shaken continuously. Bacteria were harvested by centrifugation at 2700 × G in a 5810R (Eppendorff, Rexdale, Ontario), washed and stored in aliquots at -80°C until use. Aliquots were thawed and the colony former unit (CFU) was determined before plating onto Middlebrook 7H10 plates.

### Tumor cell line

The murine transitional cell carcinoma cell line MB49 (MB49) was a kind gift from Dr. Yi Lou (University of Iowa, USA). The cells were cultured at 37°C and 5% CO_2 _in RPMI 1640 supplemented with 10% FBS (Cultilab, São Paulo, SP, Brazil), 100 U/mL penicillin and 100 μg/mL streptomycin. Tumor cells were harvested by trypsinization and suspended in RPMI 1640 without L-glutamine, FBS and antibiotics.

### Orthotopic tumor implantation

Sixty mice were placed under general anesthesia with i.p. injection of a mixture of xylazine-ketamine (0.1 mL/10 g/mouse). A 24-gauge Teflon i.v. catheter (Nipro Medical Ltda, Sorocaba, SP, Brazil) was inserted through the urethra into the bladder using an inert lubricant (sterile contact gel). To prepare the bladder for tumor implantation, a chemical lesion was induced in the bladder wall by intravesical instillation of 0.3 M AgNO_3 _(8 μL). This promoted an adequate and controlled diffuse bladder wall cauterization. After 10 seconds, the content was washed out by transurethral infusion of PBS. Then, a suspension of 2 × 10^5 ^viable tumor cells was instilled into the bladder.

### Intravesical drug administration

For Experiment I (n = 60), 24 hours after tumor implantation, intravesical BCG therapy was initiated. Mice were randomly assigned to either a control group (receiving PBS) or two treatment groups (receiving BCG or rBCG-S1PT). The BCG dose was 1 × 10^6 ^CFU/mouse once a week for one month administered periurethrally in a volume of 100 μl while the mice were lightly anesthetized. Experiment II (n = 30) was performed in the same manner as Experiment I, but the animals did not receive bladder tumor implantation.

After 35 days, the animals were sacrificed by CO_2 _inhalation, examined macroscopically for intravesical tumor (Experiment I), and bladder weight was individually verified.

### Determination of cytokine mRNA expression by qPCR

Total RNA was extracted from the bladder using Trizol^® ^(GIBCO/BRL Life Technologies, Inc.) according to the manufacturer's recommendations, and further purified with RNeasy columns (Qiagen, Valencia, CA, USA). All samples from the control, BCG and BCG-S1PT groups were co-processed to eliminate technical variations. Following DNase treatment (Sigma, St Louis, MO, USA), one microgram of RNA was reverse transcribed using SuperScript III reverse transcriptase (Invitrogen Corp., Carlsbad, CA, USA). The quantitative PCR (qPCR) primers used were: TNF-α (**F:**CATCTTCTCAAAATTCGAGTGACAA, **R:**TGGGAGTAGACAAGGTACAACCC), IL-10 (**F:**GGTTGCCAAGCCTTATCGGA, **R:**ACCTGCTCCACTGCCTTGCT) and GAPDH (**F:**CAACTCACTCAAGATTGTCAGCAA, **R:**GGCATGGACTGTGGTCATGA) (IDT, Coralville, IA, USA). qPCR was performed using an Applied Biosystems 7500 Real Time PCR System (Applied Biosystems Inc., USA). The reaction mixture contained SYBR Green PCR Master mix (Applied Biosystems), 0.5 μmol/μl l of forward and reverse primer and 1 μl of cDNA in a total volume of 10 μl. Thermocycling conditions were 50°C for 2 min., 95°C for 10 min. and 40 cycles of [95°C for 15 sec., 60°C for 1 min.]. To normalize for inefficiencies in cDNA synthesis and RNA input amounts, GAPDH mRNA expression was used. After the PCR was performed, a dissociation/melting curve was constructed in the range of 60 to 95°C. Data were analyzed using the comparative Ct method (ΔΔCt method) with the following formula: ΔCt = Ct (target) – Ct (normalized, GAPDH). The comparative ΔΔCt calculation involved finding the difference between ΔCt of the BCG and BCG-S1PT groups and the mean value of the ΔCt from control for each analyzed transcript. Fold increase in mRNA expression for the BCG groups compared to normal controls was calculated as 2^(-ΔΔCtCt) ^[13].

The RNA from the bladders from Experiment II was aggregated to perform the qPCR. The RNA extracted from bladders with tumor implantation was quantified individually.

### Histology

After gross examination the bladders were fixed in 10% buffered formalin, routinely processed with paraffin and stained by Hematoxilin & Eosin staining (HE).

### Statistical analysis

The Test of Homogeneity of Variances served as the Levene Statistic. ANOVA and Multiple Comparisons (Dunnett) were used to test for significant differences between the means. MedCalc 9.3.3.0 (MedCalc Software, Mariakerke, Belgium) was used to perform statistical analysis. Differences were considered statistically significant at *p *≤ 0.05.

## Results

### Assessment of Tumors

Considering the animals of Experiment I (n = 60) that received intravesical instillation of MB49 cells, 53 (88,3%) developed intravesical tumors after the 15-day period. At that time there was massive growth of a solid tumor inside the bladder. The mice without tumor implantation were detached from the experimental.

**Figure 1:**
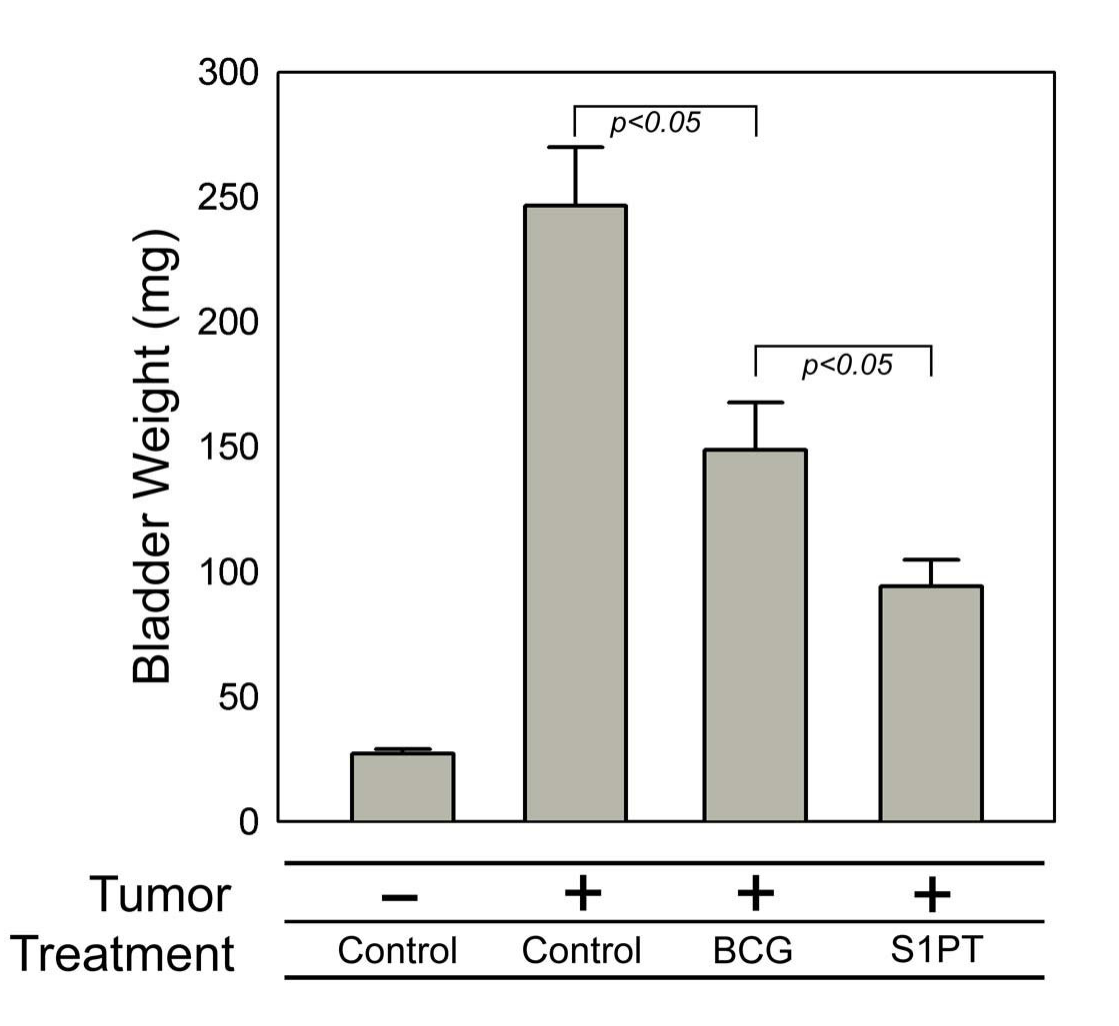
**Weight (mg) of bladders from mice instilled weekly with BCG (n = 18), S1PT (n = 18) or saline (n = 17) after four weeks of treatment.** The differences between groups were analyzed by one-way ANOVA (*p *< 0.01) and the Student-Newman-Keuls test for all pairwise comparisons after log data transformation. The mean bladder weight from Experiment I for the control group was 230 ± 110, the BCG group was 137 ± 87 and the S1PT group was 93 ± 50. From Experiment II (without MB49 tumor cell implantation), the mean bladder weight was 26.7 ± 5. *p *< 0.05 were considered statistically significant. Values were obtained from three independent experiments.

The bladders were weighed to test the potential antitumor effect of the intravesical therapy induced by the immunostimulatory action. The mean bladder weight with tumor implantation and PBS treatment was 229.8 mg (SD = 112.0 mg), while the BCG- and rBCG-S1PT-treated mice had bladders that weighed 137.2 mg (SD = 87.50 mg) and 93.70 mg (SD = 50.90 mg), respectively. The mice from Experiment II (without MB49 tumor cell implantation) had a mean bladder weight of 26.7 mg (SD = 5.0 mg). The ANOVA on log-transformed data and Student-Newman-Keuls test for all pairwise comparisons demonstrated a significant difference between the three groups (*p *< 0.001). The multiple comparisons test (Dunnett) showed significant differences between the groups treated with PBS and BCG (*p *= 0.007), PBS and rBCG-S1PT (*p *< 0.001) and BCG and rBCG-S1PT (*p *< 0.001) (Fig. 1).

**Figure 2:**
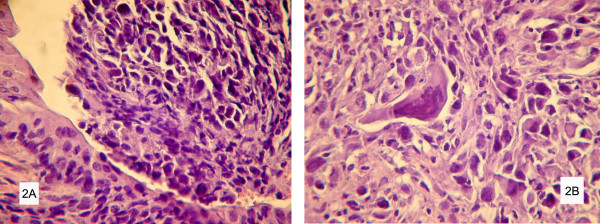
**A. Infiltrative Urothelial Carcinoma: evidence of multiple foci of lamina propria invasion HE, ×100.** B. Infiltrative Urothelial Carcinoma: infiltrating cohesive nests of cells with large hyperchromatic nuclei, HE, ×400.

Histologically, there was a high grade urothelial solid carcinoma composed of large, cubic cells, arranged in solid nests, with round, hypercromatic nuclei and one or more nucleoli. Scattered giant, unusual cells were seen in the tumor. The mitosis rate was high (50/HPF). Superficial ulceration and necrosis foci were identified. In all cases the tumor was invaded through the bladder wall, reaching the muscularis propria. No vascular invasion or perineural infiltration was observed. The transition to normal urothelium was evident, and no *in situ *carcinoma was identified (Fig. 2A, B).

### Expression of cytokine TNF-α and IL-10 mRNA by qPCR

**Figure 3:**
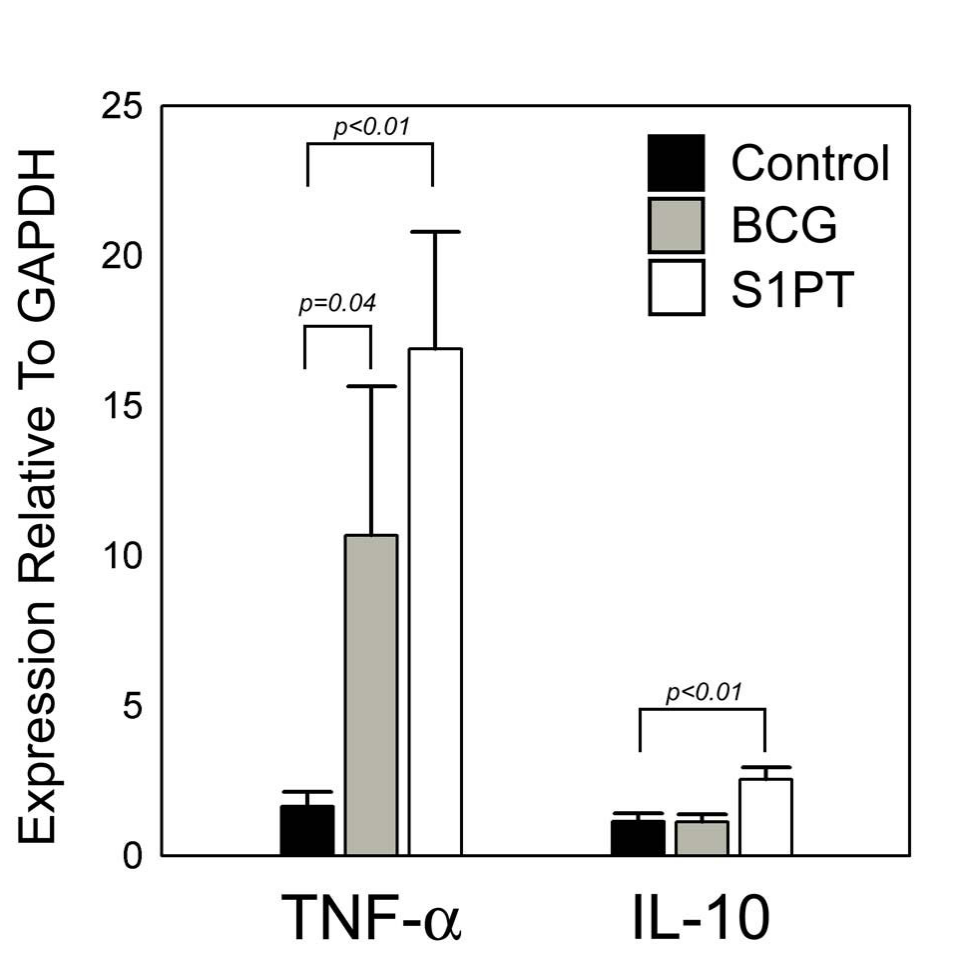
**Relative cytokine gene expression in bladders from mice challenged with MB49 tumor cells and treated with PBS (white bar), BCG (gray bar) or rBCG-S1PT (black bar), as determined by q PCR.** Data were expressed as mean values ± SEM (n = 10).

**Figure 4:**
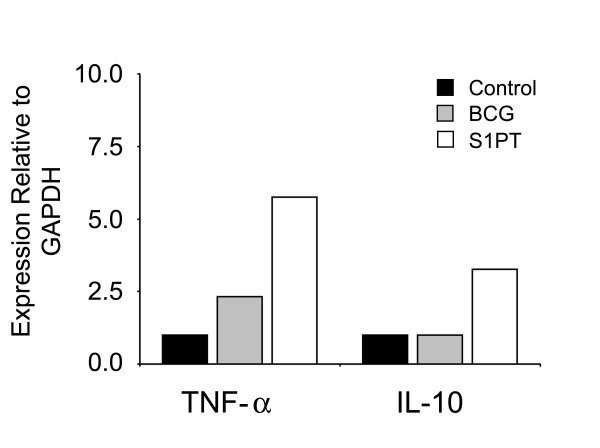
Relative cytokine gene expression in bladders from mice without tumor implantation and treated with PBS (white bar), BCG (gray bar) or rBCG-S1PT (black bar), as determined by qPCR.

In Experiment I, mice were challenged with MB49 tumor cells. Treatment with BCG and rBCG-S1PT showed significantly increased level of TNF-α, but only animals that received rBCG-S1PT showed a significantly increase in IL-10 when compared with the control group. Data were expressed as mean values ± SEM calculated from different mice (Fig. 3). Also, in Experiment II, without tumor implantation, the same patterns of expression were observed for TNF-α and IL-10 (Fig. 4).

## Discussion

The antitumor effects of the Bacillus Calmette Guerin vaccine (BCG) have long been demonstrated, but its mechanism of action still remains unclear. Bladder tumors are involved in the immune response due to their cytokine release. The experimental bladder tumor cell line MB49 does not produce IL-10, which allows examination of its levels to serve as an indicator of local stimulation. In this study, IL-10 had increased levels in treated mice, and was enhanced in the BCG-S1PT group. This interleukin is considered essential in the immune response towards a Th2 milieu and/or inhibition of Th1 cytokines. IL-10 is also important in the downregulation of delayed-type hypersensitivity (DHT) [14]. The highest TNF-α level was observed in the rBCG-S1PT group, characterizing the acute immune response pattern unleashed by *Bacillus*. We speculate that the increased level of IL-10 in this group could be a counterbalance, modulating the robust response of TNF-α. On the other hand, several animal experiments have shown diverse effects regarding the influence of IL-10 on the cancer immune response. Depending on the experimental model, IL-10 seems to favor or inhibit the existence and progression of tumors [15]. In gastric, colon, lymphomas and renal cell cancer, increased IL-10 production has been associated with a negative prognosis. Alternatively, IL-10 can inhibit the growth of new vessels within the tumor by acting on the tumor cells and indirectly by influencing infiltrating immune cells [16].

Interestingly, IL-10 knockout mice exhibit unregulated inflammatory activity exemplified by enhanced TNF-α accumulation, which is associated with a variety of pathogenic outcomes, including endotoxemia and intestinal inflammation. IL-10 is a suppressor of TNF-α synthesis and a potent anti-inflammatory cytokine that is present at the sites of tumors in a wide variety of human cancers, including transitional cell carcinoma of the bladder. We thus proposed the evaluation and comparison of the immune balance responses of TNF-α and IL-10 to rBCG-S1PT and BCG in an animal model of orthotopic bladder cancer.

rBCG-S1PT may represent a further step towards improving the Th1 immune response, especially in light of the robust TNF-α stimulation in relation to BCG alone. It has also been discovered that IL-10 is not necessary for the antitumor effect of BCG, as we observed no difference on survival between C57BL/6 wild-type and IL-10-/- mice. Thus, this Th2-related cytokine may be referred to as an immunosuppressive agent, which down-regulates cytokine production by Th1 cells [17]. The overstimulation of TNF-α in combination with the increase on IL-10 expression by rBCG-S1PT, suggests a compensatory mechanism. This would represent a counterbalance by IL-10 expression, a Th2 cytokine, as a consequence of Th1 enhancement.

These findings exhibit the adequate therapeutic effect of rBCG-S1PT by significantly reducing the mean bladder tumor weight when compared to BCG and PBS. Clearly, the combination of BCG and pertussis toxin may represent a new pathway by which other forms of rBCG may improve efficacy against bladder tumor.

## Conclusion

The data of this study indicate a significant reduction of bladder tumor volume in the rBCG group, compared to BCG and PBS, which may indicate that rBCG could be a useful substitute for wild-type BCG and suggest that the potential modulation between TNF-α and IL-10 cytokine production may have therapeutic value.

## Competing interests

The authors declare that they have no competing interests.

## Consent section

Not applicable (experimental study)

## Authors' contributions

DCC carried out the tumor implantation, intravesical drug administration, molecular genetic studies, and drafted the manuscript. PMA participated in the tumor implantation, intravesical drug administration, molecular genetic studies, and performed the statistical analysis. RCB participated in the molecular genetic studies and reviewed the statistical analysis. IPN participated in the molecular genetic studies. FEV participated in the molecular genetic studies. LCCL participated in its design and coordination. EA participated in the molecular genetic studies. KRL participated in the study coordination and performed the histological analysis. MS coordinated the study. All authors read and approved the final manuscript.
